# Clinical characteristics of dry eye with ocular neuropathic pain features: comparison according to the types of sensitization based on the Ocular Pain Assessment Survey

**DOI:** 10.1186/s12886-020-01733-1

**Published:** 2020-11-18

**Authors:** Jonghwa Kim, Hyeon Jeong Yoon, In Cheon You, Byung Yi Ko, Kyung Chul Yoon

**Affiliations:** 1 Department of Ophthalmology, Chonnam National University Medical School and Hospital, Gwangju, South Korea; 2 Department of Ophthalmology, Research Institute of Clinical Medicine of Chonbuk National University-Biomedical Research Institute of Chonbuk National University Hospital, Jeonju, South Korea; 3 Department of Ophthalmology, Konyang University Hospital and College of Medicine, Daejeon, South Korea

**Keywords:** Ocular pain, Ocular neuropathic pain, Neuropathic corneal pain, Neuropathic ocular pain, Dry eye, Sensitization

## Abstract

**Background:**

To compare the clinical characteristics of dry eye patients with ocular neuropathic pain features according to the types of sensitization based on the Ocular Pain Assessment Survey (OPAS).

**Methods:**

Cross-sectional study of 33 patients with dry eye and ocular neuropathic pain features. All patients had a comprehensive ophthalmic assessment including detailed history, the intensity and duration of ocular pain, the tear film, ocular surface, and Meibomian gland examination, and OPAS. Patients with < 50% improvement in pain intensity after proparacaine challenge test were assigned to the central-dominant sensitization group (central group) and those with ≥50% improvement were assigned to the peripheral-dominant sensitization group (peripheral group). All variables were compared between the two groups.

**Results:**

No significant differences were observed in age, sex, underlying diseases, history of ocular surgery, duration of ocular pain, tear film, ocular surface and Meibomian gland parameters (all *p* > 0.05). Ocular pain and non-ocular pain severity and the percentage of time spent thinking about non-ocular pain were significantly higher in the central group than in the peripheral group (all *p* < 0.05). Central group complained more commonly of a burning sensation than did the peripheral group (*p* = 0.01).

**Conclusions:**

Patients with central-dominant sensitization may experience more intense ocular and non-ocular pain than the others and burning sensation may be a key symptom in those patients.

## Background

Dry eye disease (DED) is a multifactorial disease and the management of DED is often complicated because the disease varies from patients to patients, both in severity and in character [1]. DEWS II report has classified ocular neuropathic pain as another entity differentiated from DED [2]. However, some studies reported that patients diagnosed with dry eye often describe features of neuropathic pain, including spontaneous pain, dysesthesias, allodynia, and hyperalgesia [3, 4]. It is established that ongoing damage to the corneal surface and nerve endings induced by tear film instability and persistent inflammation can cause peripheral neuronal sensitization, and repeated peripheral nerve injury can lead to central neuronal sensitization [5–9]. Topical anesthetic may be insufficient to alleviate pain in patients with centralized ocular neuropathic pain. Crane et al. [10] have introduced the proparacaine challenge test which can discriminate if there is a centralized component in ocular pain.

There is no gold standard for diagnosing ocular neuropathic pain. Owing to the scarcity of available signs, the diagnosis mainly depends on clinical history, symptoms, and ophthalmologic examination results [11, 12]. Many studies attempted to evaluate patients with ocular neuropathic pain with dry eye related questionnaires, such as the Ocular Surface Disease Index, Dry Eye Questionnaire, and Dry Eye-Related Quality-of-Life Score [13–15]. The Ocular Pain Assessment Survey (OPAS) is a validated questionnaire for ocular pain and assesses non-ocular pain, quality of life (QoL), aggravating factors and associated factors as well [16].

In this study, we collected clinical data and the OPAS questionnaires from three eye centers to investigate the characteristics of patients with DED and ocular neuropathic pain features, the association of ocular neuropathic pain features with pre-existing medical conditions, and the clinical differences between groups classified according to the dominant types of sensitization.

## Methods

A multicenter, cross-sectional study was performed between January 2, 2018 and June 30, 2019, at the outpatient departments of three eye centers in Korea, namely, Chonnam National University Hospital, Konyang University Hospital, and Chonbuk National University Hospital. Informed consent was obtained from each patient. Ethical approval was obtained from the ethical committees of all participating hospitals, and the study protocol followed the guidelines of the Declaration of Helsinki.

### Patients and groups

Patients complaining of continuous severe ocular pain or burning sensation of pain score 7 or more using the Wong-Baker FACES® Pain Rating Scale with little or no corneal staining were included. The diagnosis of DED was made based on DEWS II criteria [2]. Patients who complained of ocular discomfort and had tear film break-up time (TBUT) less than 10 s were included. Patients with active inflammation of ocular surface and eye lid, orbital diseases that could induce pain, glaucoma, and migraine were excluded. Patients with deficient tear secretion with Schirmer test scores less than 5 mm/5 min without anesthesia were also excluded.

Patients were divided into two subgroups according to their response to the proparacaine challenge test [6, 10]. As part of the test, 10 μL of 0.5% proparacaine hydrochloride (Alcaine, Alcon, Fort Worth, TX, USA) was instilled in the inferior fornix of each eye. The Wong-Baker FACES® Pain Rating Scale scores (range, 0–10) were recorded before and 15 s after proparacaine administration. Patients with more than a 50% decrease in pain scores after 15 s were assigned to the peripheral-dominant sensitization group (peripheral group) and those with a decrease in pain scores equal to or less than 50% were assigned to the central-dominant sensitization group (central group). Information on demographics and thorough history of systemic diseases and ocular surgery was collected for each patient.

### Tear film, ocular surface, and Meibomian gland assessment

TBUT, Schirmer test score, and corneal staining score (CSS) were evaluated by three cornea specialists (K.C.Y, I.C.Y, and B.Y.K) at the first visit. TBUT was assessed three times after the instillation of fluorescein dye, and the mean TBUT recorded in seconds was used for analysis. CSS was evaluated subsequently by employing a white light and cobalt blue filter, using the area-density index, scoring the area (0–3) and density (0–3) of the superficial punctate corneal lesion, and multiplying the area and density scores (0–9) [17]. The Schirmer test was performed using a calibrated sterile strip (Color Bar Schirmer Tear Test, Eagle Vision Inc., Memphis, TN, USA) under topical anesthesia (0.5% proparacaine hydrochloride). The sterile strips were placed in the lateral canthus, away from the cornea, for 5 min with the eyes closed. Schirmer test scores were recorded in millimeters of wetting after 5 min.

The Meibomian gland (MG) expressibility was assessed by applying digital pressure onto the lower central eyelid and counting the number of expressed gland orifices within the central eighth of the lower eyelid and was scored on a 0–3 grading scale [18]. MG secretion quality score was also assessed using a 0–3 grading scale [18]. The eye with worse pain was chosen for statistical analysis for each patient because the OPAS questions are conducted on the eye with more pain [16]. When both eyes had the same pain intensity, the values from the right eye were included in the analysis.

### Ocular pain assessment

All patients completed the OPAS, which is a validated questionnaire for neuropathic pain that combines patient responses regarding ocular and non-ocular pain intensity, impact on QoL, aggravating factors, associated factors, and symptomatic relief [16]. The questions were divided into sections for analysis: questions 4–9 pertained to the intensity of ocular pain; questions 10–12, non-ocular pain; questions 13–19, the QoL; questions 20–21, aggravating factors; and questions 22–25, associated factors. After excluding the section on symptomatic relief, only questions 4–25 were analyzed in this study.

### Statistical analysis

SPSS Statistics for Windows, Version 23.0 (IBM Corp., Armonk, NY, USA) was used for statistical analysis. All data are shown as the mean ± standard deviation. The Kolmogorov-Smirnov test was performed for continuous variables, and the normally distributed variables were age, OPAS ocular pain intensity score, impairment in walking score, pain-associated redness, and burning sensation score. The independent t-test was used to identify between-group differences in the mean values of these variables. For other variables that were not normally distributed, the Mann-Whitney U-test was performed. Pearson’s correlation analysis between the Wong-Baker FACES® Pain Rating Scale and the ocular pain severity score of OPAS was conducted. A *p* value < 0.05 was considered statistically significant. The sample size of 17 subjects in the central group and 16 subjects in the peripheral group provided approximately 88% of power to show a significant difference with the independent t-test.

## Results

A total of 33 patients were analyzed. On the basis of the proparacaine challenge test results, 17 patients were assigned to the central group and 16 patients to the peripheral group; their mean ages were 59.12 ± 11.58 and 58.13 ± 12.86 years, respectively. There were more women than men in both groups. Table 1 shows the demographic features and personal history of the patients. Two patients in the central group and one in the peripheral group had been diagnosed with chronic pain syndrome (CPS). In the central group, two patients had psychological disorders and two others had neurological disorders, whereas in the peripheral group, none had psychological or neurological disorders. Six patients in the central group and three in the peripheral group had previous cataract surgery. No significant differences were observed in the demographic and personal history data between the two groups (all *p* > 0.05).

**Table 1:** Demographics of dry eye patients with ocular neuropathic pain features

	All	Types of sensitization	
		Central (***N*** = 17)	Peripheral (***N*** = 16)
Demographics			
Age (years) ^a^	58.64 ± 12.03	59.12 ± 11.58	58.13 ± 12.86
Sex (M:F)	12: 21	6:11	6:10
Comorbidities, n (%)	18 (54.5)	12 (70.6)	6 (37.5)
Hypertension	9 (27.3)	4 (23.5)	5 (31.3)
Chronic pain syndrome	3 (3.1)	2 (11.8)	1 (6.3)
Diabetes mellitus	2 (6.1)	2 (11.8)	0 (0)
Neurologic disorder	2 (6.1)	2 (11.8)	0 (0)
Psychological disorder	2 (6.1)	2 (11.8)	0 (0)
Ocular Surgery, n (%)	12 (36.4)	8 (47.1)	4 (25.0)
Cataract surgery	9 (27.3)	6 (35.3)	3 (18.8)
LASIK/LASEK	1 (3.0)	0 (0)	1 (6.3)
Others	2 (6.1)	2 (11.8)	0 (0)

^a^Values are presented as mean ± SD

The mean values for TBUT, Schirmer test score, CSS, and MG quality and expressibility in patients with ocular neuropathic pain were 4.67 ± 2.01 s, 7.22 ± 5.09 mm, 0.42 ± 0.75, 1.21 ± 0.82, and 0.61 ± 0.79, respectively. No significant differences were found in tear film, ocular surface, and MG parameters between the central and peripheral groups (all *P* > 0.05) (Table 2). The duration of ocular pain was 38.35 ± 31.37 months in the central group and 36.00 ± 30.54 months in the peripheral group but the difference between groups was not significant (*p* = 0.69).

**Table 2:** Tear film, ocular surface and meibomian gland status in dry eye patients with ocular neuropathic pain features according to the dominant type of sensitization

	All	Types of sensitization		
		Central (***N*** = 17)	Peripheral (***N*** = 16)	***p***-value
Tear film and ocular surface parameters				
TBUT (sec)	4.67 ± 2.01	4.41 ± 1.77	4.94 ± 2.26	0.46
Schirmer test score (mm)	7.22 ± 5.09	6.82 ± 2.81	7.56 ± 6.70	0.68
Corneal staining score (0–9)	0.42 ± 0.75	0.38 ± 0.77	0.43 ± 0.79	0.41
MG parameters				
MG quality (0–3)	1.12 ± 0.82	1.08 ± 0.76	1.00 ± 1.00	0.98
MG expressibility (0–3)	0.61 ± 0.79	0.54 ± 0.78	0.43 ± 0.79	0.76

Data presented as mean ± SD. *TBUT* Tear break-up time, *MG* Meibomian gland.

Table 3 summarizes the OPAS scores in the participants. The ocular pain severity score was significantly higher in the central group (36.71 ± 11.48) than in the peripheral group (25.06 ± 11.21) (*p* < 0.01). The pain scale assessed by the Wong-Baker FACES® Pain Rating Scale was also significantly higher in the central group (7.06 ± 2.33 vs. 5.19 ± 2.56, *p* = 0.04) and the Pearson’s correlation coefficient between the Wong-Baker FACES® Pain Rating Scale and the OPAS score was 0.78 (*p* < 0.001). Non-ocular pains, such as headache, backache, and arthralgia, scored higher in the central group (6.12 ± 3.12) than in the peripheral group (3.81 ± 2.90, *p* = 0.04), and patients in the central group spent more time thinking about non-ocular pain than did those in the peripheral group (0.56 ± 0.35 vs. 0.27 ± 0.26) (*p* = 0.01). The scores regarding QoL were highest for reading impairment in both groups, but no significant differences were observed between groups. Although the difference was not statistically significant, most of the QoL scores were higher in the central group (reading, driving, walking, mood, and social activity) than in the peripheral group. The aggravating factors were scored by the percentage of worsening of ocular pain according to mechanical stimuli, such as wind, dry air, heat, and air conditioning, and chemical stimuli, such as volatile chemicals, fumes, and cosmetic fragrances. The associated factors were scored as a percentage of the frequency with which each symptom was accompanied by ocular pain. The percentage values were later divided by 100 for analysis. The values of aggravating and associated factors did not differ significantly between the two groups, except for burning sensation (0.43 ± 0.32 in the peripheral group and 0.73 ± 0.34 in the central group; *p* = 0.01).

**Table 3:** Comparison of Ocular Pain Assessment Survey results between the central-dominant and peripheral-dominant sensitization groups

	Types of sensitization		
	Central (***N*** = 17)	Peripheral (***N*** = 16)	***p***-value
Ocular and non-ocular pain			
Pain severity (0–60)	36.71 ± 11.48	25.06 ± 11.21	< 0.001
Pain other than the eyes (0–10)	6.12 ± 3.12	3.81 ± 2.90	0.04
Time spent thinking about non-eye pain (0–1)	0.56 ± 0.35	0.27 ± 0.26	0.01
Quality of life			
Reading	7.50 ± 1.73	5.33 ± 3.08	0.25
Driving	6.83 ± 2.17	4.43 ± 3.31	0.11
Walking	4.31 ± 3.68	3.00 ± 2.08	0.27
Mood	6.23 ± 2.98	3.57 ± 3.41	0.11
Sleep	3.23 ± 3.06	3.71 ± 3.87	0.36
Social activity	5.69 ± 2.78	2.71 ± 3.04	0.20
Aggravating factors (0–1)			
Mechanical stimuli	0.53 ± 0.32	0.47 ± 0.39	0.35
Chemical stimuli	0.55 ± 0.37	0.36 ± 0.34	0.85
Associated factors (0–1)			
Redness	0.28 ± 0.22	0.44 ± 0.45	0.84
Burning sensation	0.73 ± 0.34	0.43 ± 0.32	0.01
Photophobia	0.72 ± 0.23	0.43 ± 0.39	0.59
Tearing	0.67 ± 0.33	0.27 ± 0.29	0.74

Data presented as mean ± SD.

## Discussion

In the present study, dry eye patients with ocular neuropathic pain features were divided into two groups on the basis of proparacaine challenge test results. Although clinical parameters associated with the tear film and ocular surface, such as TBUT, basal tear secretion, CSS, and MG parameters, were not significantly different between the two groups, the central group complained of more severe ocular pain and non-ocular pain than did the peripheral group. In addition, we noticed that patients in the central group complained of a burning sensation more commonly than did those in the peripheral group.

The underlying mechanisms of DED is ocular surface inflammation and damage induced by tear hyperosmolarity [1]. Tear hyperosmolarity can be a direct cause of ocular discomfort and it can also lead to a death of epithelial cells and a loss of goblet cells and induce ocular discomfort indirectly [1, 19]. However, some patients may suffer from allodynia, hyperalgesia, hypesthesia, and hyperesthesia without any obvious abnormal findings and are thought to have ocular neuropathic pain features [20, 21]. Ocular neuropathic pain is known to be associated with other systemic diseases such as depression, anxiety, fibromyalgia and headache [6]. It is more frequently reported in females than males [22].

It is well established that structural and functional changes occur in ocular surface sensory nerves in DED. Reduced tear secretion causes stress on ocular mucosal epithelium, leading to local inflammation and peripheral nerve damage [23]. Long-term inflammation and nerve injury alter trigeminal ganglion and brainstem neurons, changing their excitability, connectivity, and impulse firing causing dysesthesias and neuropathic pain referred to the ocular surface [23–25]. Subcategorizing DED patients based on peripheral and/or central dysfunction has important implications in the treatment the disease [26]. Patients with peripheral abnormalities may benefit from treatments targeting ocular surface inflammation and hyperosmolarity whereas patients with neuropathic pain features may need more centrally acting neuromodulators [27].

In vivo confocal microscopy (IVCM) visualizes microstructures, including corneal nerve plexus [28–30]. Some studies have demonstrated the usefulness of the IVCM in evaluating corneal neuropathies by visualizing the decrease in sub-basal corneal nerve density, increase in nerve tortuosity, activation of keratocytes and spindle in corneal stroma, and the presence of microneuromas in the stroma [11, 29, 30]. In many general clinical settings, the IVCM is not available for evaluation.

The 0.5% proparacaine challenge test is a useful method to assess the central sensitization of ocular pain in a general clinical setting which does not provide research equipments such as esthesiometry or confocal microscopy [10]. In previous studies, patients with complete relief of pain after proparacaine administration were classified into the peripheral sensitization group, those with consistent pain without relief were classified into the central sensitization group, and those with partial relief were classified into the mixed sensitization group [10]. Dieckmann et al. [6] reported that most of the patients showed partial improvement in pain, suggesting that central sensitization and peripheral sensitization were mixed, and the rate of contribution to pain depends on the etiology or duration of the disease. Similarly, in our study, 27 out of 33 (81.82%) patients showed mixed sensitization. Therefore, for the analysis, we divided the patients into two groups: central-dominant sensitization group with less than 50% improvement in pain, and peripheral-dominant sensitization group with 50% or more improvement in pain. No significant differences were observed in pain duration between the two groups. Comorbidities such as CPS, neurologic disorders, and psychological disorders were more common in the central group (70.6%) than in the peripheral group (37.5%).

Previous studies have attempted to evaluate patients with ocular pain by using dry eye-related questionnaires, such as the Ocular Surface Disease Index, Dry Eye Questionnaire, and Dry Eye–Related Quality-of-Life Score [13–15]. In many of these questionnaires, ocular pain and dry eye symptoms are queried simultaneously. The Neurobehavioral Rating Scale, which is used as a primary outcome measure for chronic pain, and the Neuropathic Pain Symptom Inventory (NPSI), which is used to evaluate neuropathic pain, have also been used for the evaluation of ocular pain [31, 32]. Recently, the NPSI was appropriately adapted for evaluating ocular pain, and a modified NPSI-Eye was later developed during our study period [33]. Among the available questionnaires, we used the OPAS to evaluate patients with ocular neuropathic pain features, because it provides multi-dimensional information not only on the severity of ocular pain but also on associated and aggravating factors and the QoL; moreover, it is a validated questionnaire for ocular pain [16]. The Pearson’s correlation analysis between the Wong-Baker FACES® Pain Rating Scale and the OPAS score showed good correlation between two measures.

Kalangara et al. [5] used the term “burning eye syndrome” for a subset of dry eye representing a neuropathic pain of the eye. Burning sensation is often diagnosed as neuropathic pain when primary painful conditions are excluded. Burning mouth syndrome (BMS) shares many features with ocular neuropathic pain [34–36]. BMS is characterized by abnormal burning sensation in the oral cavity, but in the absence of clinical lesions. Patients diagnosed with BMS have been reported to have psychiatric disorders, such as depression and anxiety, or are mentally vulnerable to stress. In addition, loss of small-diameter nerve fibers in the oral mucosa and decreased brain activation to heat stimuli on functional magnetic resonance imaging have been demonstrated in these patients [36]. Our finding that patients with central-dominant sensitization complained more commonly of a burning sensation may support the association between BMS and ocular neuropathic pain. Further investigations focusing on both the oral mucosal and corneal nervous systems could help identify the underlying mechanism of ocular neuropathic pain.

Our study has several limitations. First, the sample size may not be enough to prove the clinical significance of results. Further studies with larger sample sizes that provide greater power could advance the results of this study. Second, because this study is a multicenter study, subtle bias may be present in conducting clinical examinations. Third, corneal esthesiometry or IVCM imaging were not obtained in this study. Fourth, we used an arbitrary 50% cut-off value in the proparacaine challenge test for the analysis and it might not be a validated value for determining the types of sensitization. Further studies with more precise diagnostic equipment are warranted in the future.

## Conclusion

In conclusion, dry eye patients with central-dominant sensitization may experience more intense ocular and non-ocular pain than the others. Burning sensation may be a key symptom in ocular neuropathic pain. The OPAS questionnaire can be a good option for evaluating whether patients have ocular neuropathic features. Further investigations on corneal nervous system and peripheral and central sensitization associated with dry eye may give clue to the management of patients with ocular neuropathic pain features.

## Data Availability

Data supporting our findings are contained in the manuscript. However, the raw data set on which the conclusion was made is available on request from Professor Kyung Chul Yoon (contact email: kcyoon@jnu.ac.kr).

## Abbreviations

DED: Dry eye disease
OPAS: Ocular pain assessment survey
QoL: Quality of life
TBUT: Tear film break-up time
CSS: Corneal staining score
MG: Meibomian gland
CPS: Chronic pain syndrome
IVCM: In vivo confocal microscopy
BMS: Burning mouth syndrome
